# Supplementary material for: Nanoscale architecture and dynamics of CaV1.3 channel clusters in cardiac myocytes revealed by single channel nanoscopy
Source: J Mol Cell Cardiol Plus. 2025 Oct 16;14:100490. doi: 10.1016/j.jmccpl.2025.100490 (PMC12580110; doi:10.1016/j.jmccpl.2025.100490)

Supplementary Materials

"Nanoscale architecture and dynamics of $Ca_V1.3$ channel clusters in cardiac myocytes revealed by single channel nanoscopy"

1) Supplementary Software (8 files, Matlab and ImageJ macro language) for quantitative image analysis workflows employed in Fig. 1 – 5.

Software files correspond to experiments of the main text figures as follows:

Fig. 1: „ImageJ_1 STED CaV cluster analysis.ijm".
Fig. 2: „DNA_PAINT_1_DME_drift_correction.m";
„DNA_PAINT_2_MolecularMapping.m";
„DNA_PAINT_3_ClusterAnalysis.m".
Fig. 3: „SPT_1_Diffusion_analysis_trackit.m";
„SPT_2_SimulationImmobileLocError".
Fig. 4: „ImageJ_2 Confocal CRU colocalization.ijm".
Fig. 5: „ImageJ_3 Confocal CTT cluster thresholding.ijm".

Functionality is further described in the Methods section and the in-file comments.

2) Supplementary Figures S1 – S8: see below

Figure S1: Automated patch clamp measurements confirm similar electrophysiological characteristics of wild-type and Halo- or GFP-tagged $Ca_V1.3$ channels expressed in HEK293 CT6232 cells.

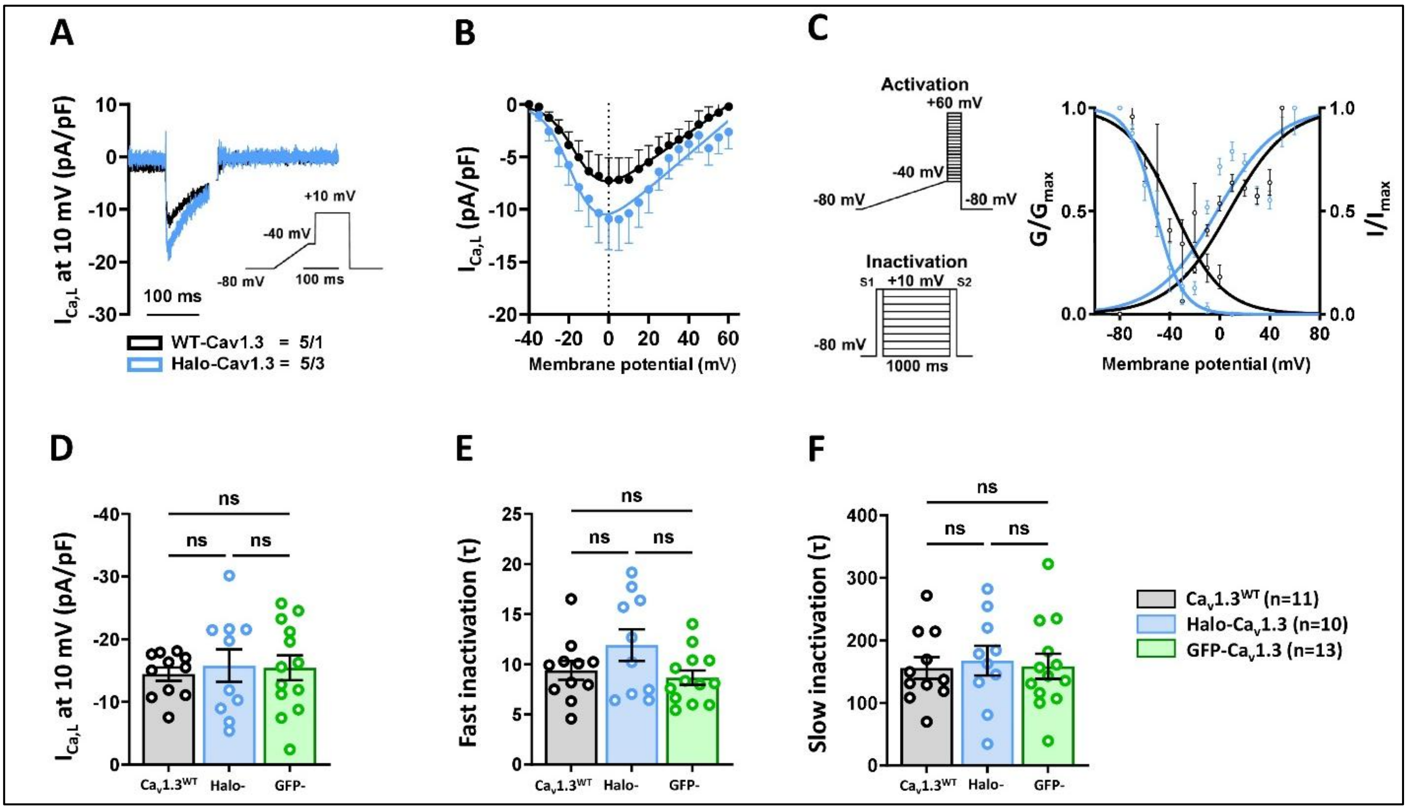

HEK293 CT6232 cells expressing the accessory Ca_V channel subunits $\alpha_2\delta_1$ and β_3 were induced to express pore-forming, wild-type α_{1D} ($Ca_V1.3^{WT}$) or transfected with a plasmid encoding the tagged α_{1D} (Halo-$Ca_V1.3$). Whole-cell calcium currents were measured using the Nanion SyncroPatch 384 device.

In a first set of experiments Halo-tagged $Ca_V1.3$ channels were compared to WT channels **(A-C)**, showing similar electrophysiological characterisics: **A,** $Ca_V1.3^{WT}$ and Halo-$Ca_V1.3$ cells show typical $I_{Ca,L}$ membrane current (I_M) with the shown voltage-ramp protocol. **B,** Current–voltage (I–V) relationship curves for $I_{Ca,L}$ WT-$Ca_V1.3$ and Halo-$Ca_V1.3$ cells have similar shapes, while different amplitudes indicate different expression levels. **C,** $Ca_V1.3^{WT}$ and Halo-$Ca_V1.3$ cells showing similar current activation and inactivation. Note the voltage-ramp protocols for I–V/activation experiments and S1/S2 voltage protocols for $I_{Ca,L}$ inactivation. The graph shows similar $I_{Ca,L}$ activation (G/G_{max}) in $Ca_V1.3^{WT}$ and Halo-$Ca_V1.3$, with corresponding inactivation (I/I_{max}) curves. (n = number of WT-$Ca_V1.3$ cells, 5 from 1 batch, and Halo-$Ca_V1.3$ cells, 5 from 3 batches. The I–V curves were fitted with a modified Boltzmann equation.)

In a second set of experiments both GFP- and Halo-tagged $Ca_V1.3$ channels were compared to WT channels **(D-F)**, with similar electrophysiological characterisics: Peak $I_{Ca,L}$ amplitudes (pA/pF) (**D**) and biphasic inactivation kinetics of $I_{Ca,L}$ analyzed as fast inactivation (**E**) and slow inactivation (**F**) were not statistically different (ns). All parameters were compared using ANOVA Kruskal-Wallis test with Dunn's correction, n = number of cells, data indicate mean ± SEM.

Figure S2: Brightness referencing method for molecular counting of JF646 fluorophores.

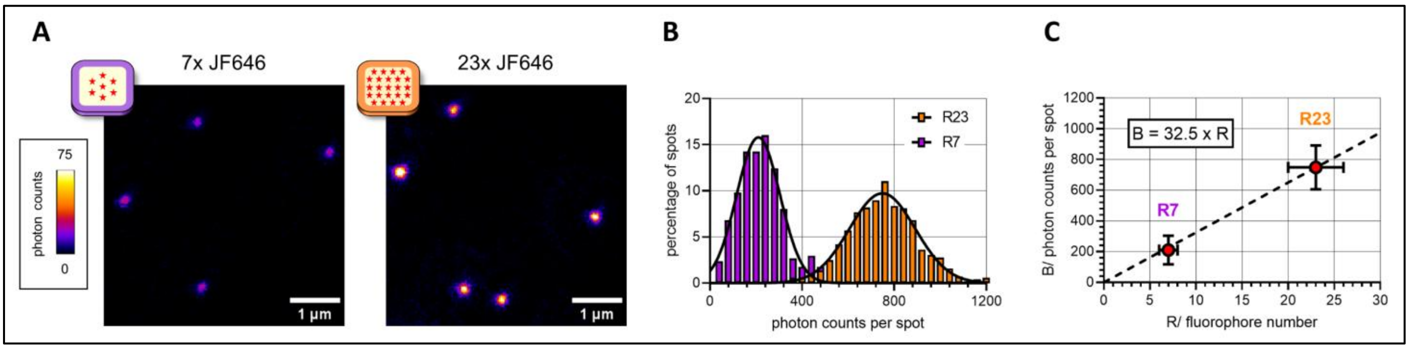

DNA Origami linked to 7 or 23 JF646 dye molecules were immobilized on coverslips and recorded by STED imaging under equal conditions as for cellular Halo-$Ca_V1.3$ cluster imaging **(A)**. By image analysis of spot-like signals, a distribution of integrated photon counts across all detected spots was determined for each sample **(B)**. The histograms were fitted by normal distributions to retrieve mean brightness values, which was used for a linear fit of spot brightness to dye molecule counts in **(C)**. The determined conversion factor (32.5 photon counts per fluorophore) was used for image analysis of Halo-$Ca_V1.3$ samples to retrieve labeled channel counts within clusters.

Figure S3: Optimization of DNA-PAINT image reconstruction and DBSCAN clustering.

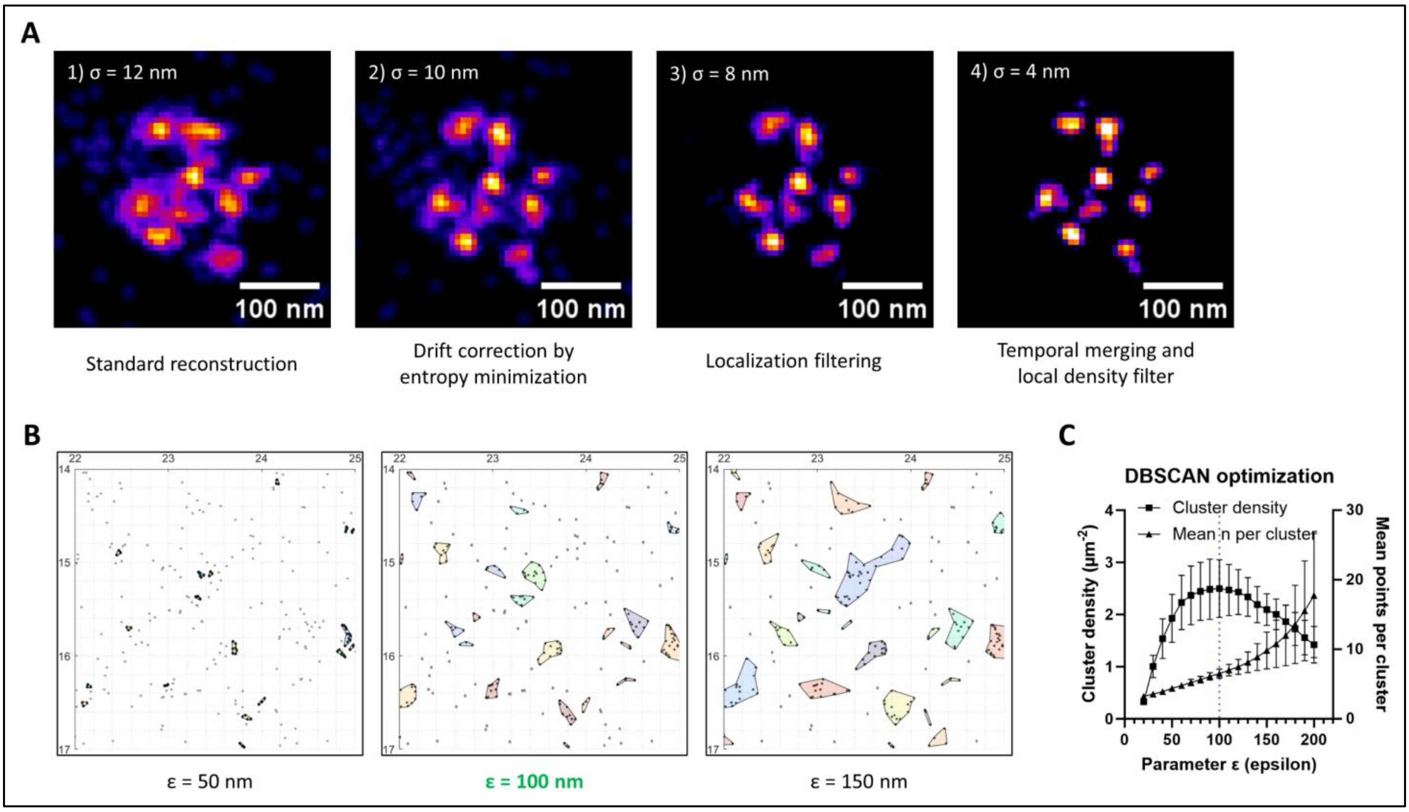

A) Representative reconstruction of a GFP-Ca$_V$1.3 channel cluster imaged by DNA-PAINT in TIRF mode. A successive improvement of the localization-based image reconstruction over standard reconstruction (1) was achieved by applying a customized version of drift correction by entropy minimization (DME, 2), followed by either histogram-based localization filtering (3), or followed by temporal merging of subsequent localizations and local density filtering (4).

B) The point clustering algorithm DBSCAN was applied to DNA-PAINT molecular map data. Three exemplary values for the parameter ε give rise to distinct clustering results.

C) Graph showing the change in DBSCAN cluster density and points per cluster as a function of ε parameter values at minPts = 3. For ε = 100 nm, the highest cluster density (2.5 µm^{-2}) is observed, while higher ε values lead to merging of pre-existing clusters and increased heterogeneity.

Figure S4: Confocal timelapse imaging demonstrates immobility of Halo-Ca_V1.3 clusters across time scales.

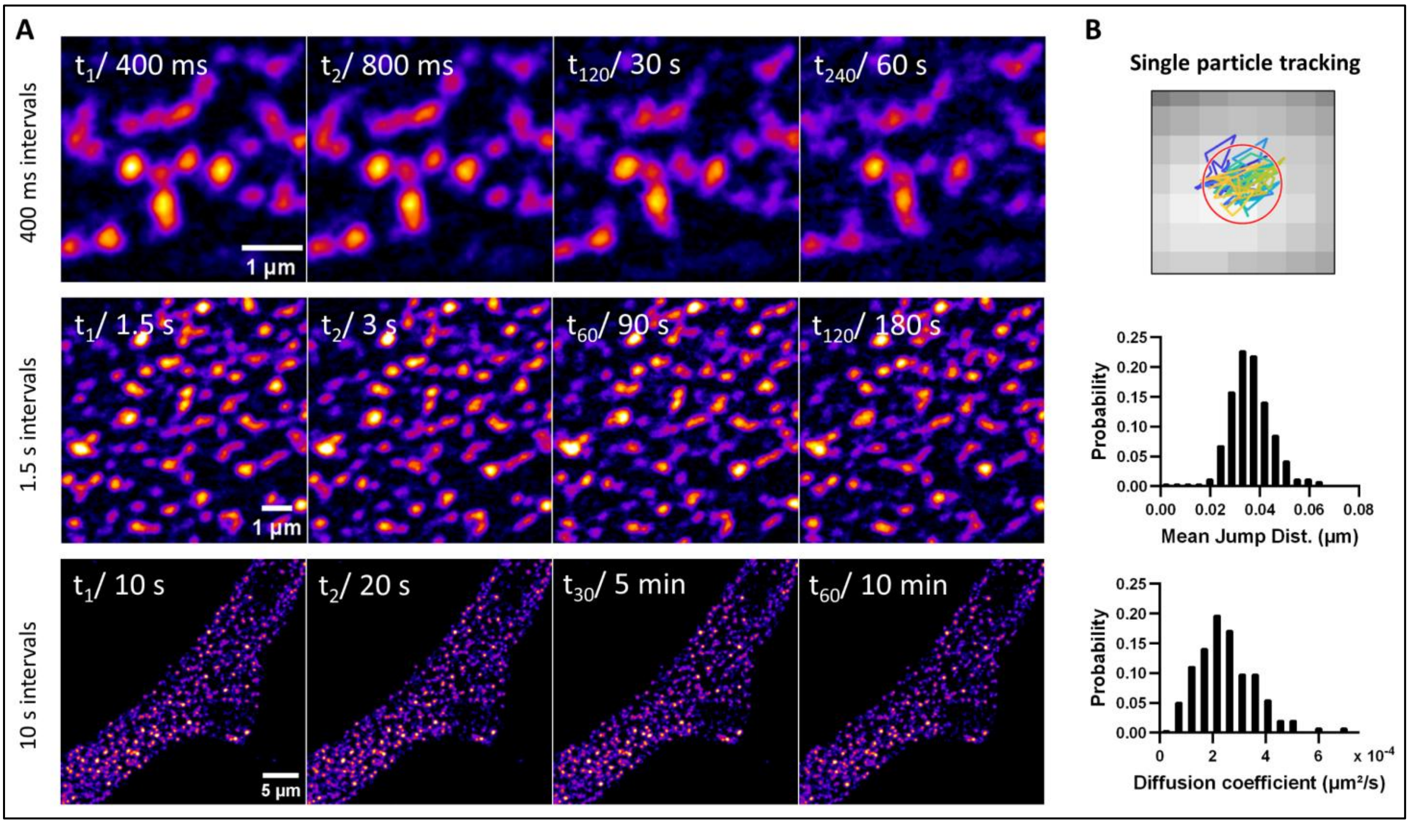

A) Confocal timelapse imaging of hiPSC-aCM expressing Halo-Ca_V1.3 (row 1+2) or GFP-Ca_V1.3 (row 3) shows cluster positions in the basal plasma membrane. Images series were recorded in intervals of 400 ms, 1.5 s and 10 s. For each timelapse, the first, second, middle and last frame are shown.

B) Representative trajectory of a single cluster position shown on a 30 nm pixel grid, generated by SPT of a timelapse at 1.5 s intervals and 30 nm pixel size. Quantitative analysis of SPT data reveals low jump distances of ~ 35 nm reflecting the localization uncertainty and MSD fit-derived diffusion coefficients of less than 10^{-4} µm²/s, thus confirming immobility of the tracked cluster positions.

Figure S5: Supporting data for single particle tracking analysis.

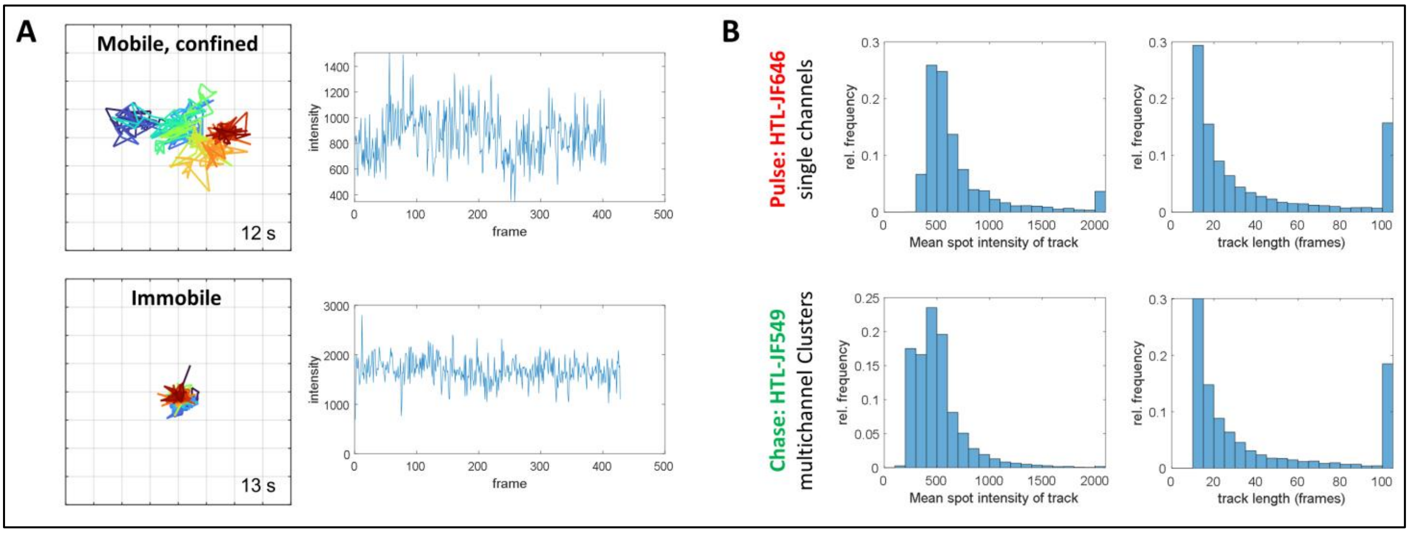

A) Intensity time traces for the exemplary tracked spots shown in Fig. 3C. No bleaching steps were observed in the majority of long tracks.

B) Mean spot intensity and track length distributions indicative of tracking performance were calculated. Both metrics show similar distributions for both imaging modes, which excludes a potential bias in the comparative diffusion analysis.

Figure S6: Halo-Ca_V1.3 colocalization with nanodomain and compartment markers.

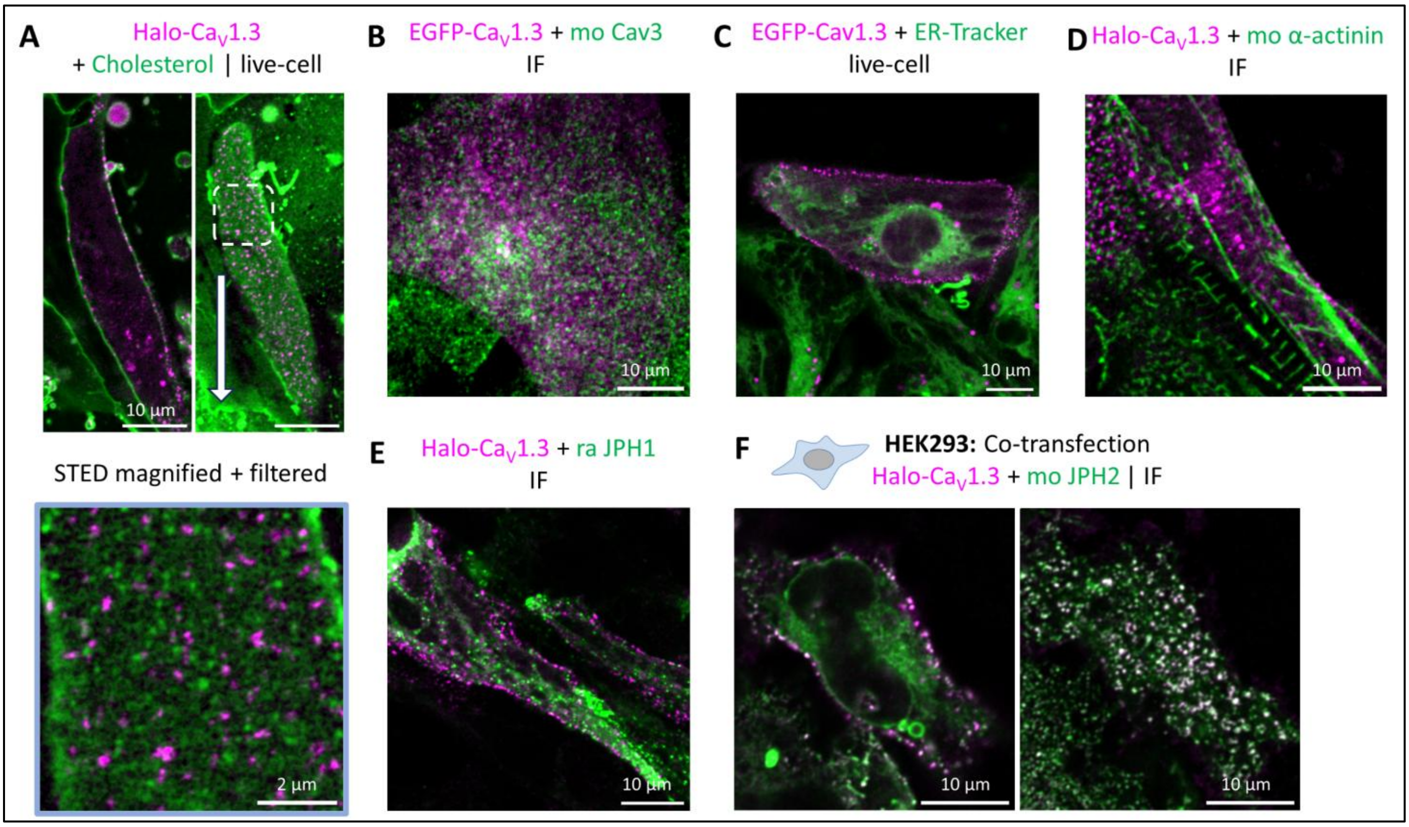

A) Live-cell confocal images of hiPSC-aCM expressing Halo-Ca_V1.3, labeled by HTL-JF646 and Cholesterol-StarOrange. Ca_V1.3 clusters and Cholesterol both localized to the plasma membrane (top), but dual-channel STED imaging in the basal membrane focal plane (bottom) showed rather exclusion-like arrangement with Cholesterol-containing nanodomains.

B) Immunofluorescence of hiPSC-aCM expressing EGFP-Ca_V1.3 showed rather low colocalization with Caveolin-3 (Cav3).

C) Live-cell imaging of hiPSC-aCM showed a mutually exclusive distribution of EGFP-Ca_V1.3 and endoplasmic reticulum, labeled by ER-Tracker Red.

D) Immunofluorescence of hiPSC-aCM expressing Halo-Ca_V1.3 showed no colocalization with cardiac α-actinin or Junctophilin-1 (JPH1) **(E)**.

F) Immunofluorescence of HEK293 CT6232 cells transfected with Halo-Ca_V1.3 and JPH2-CFP showed extensive colocalization of clustered spots in the basal membrane focal plane.

Figure S7: $Ca_V1.3$ C-terminal construct expression in HEK293 leads to cluster formation independent of the cardiac proteome.

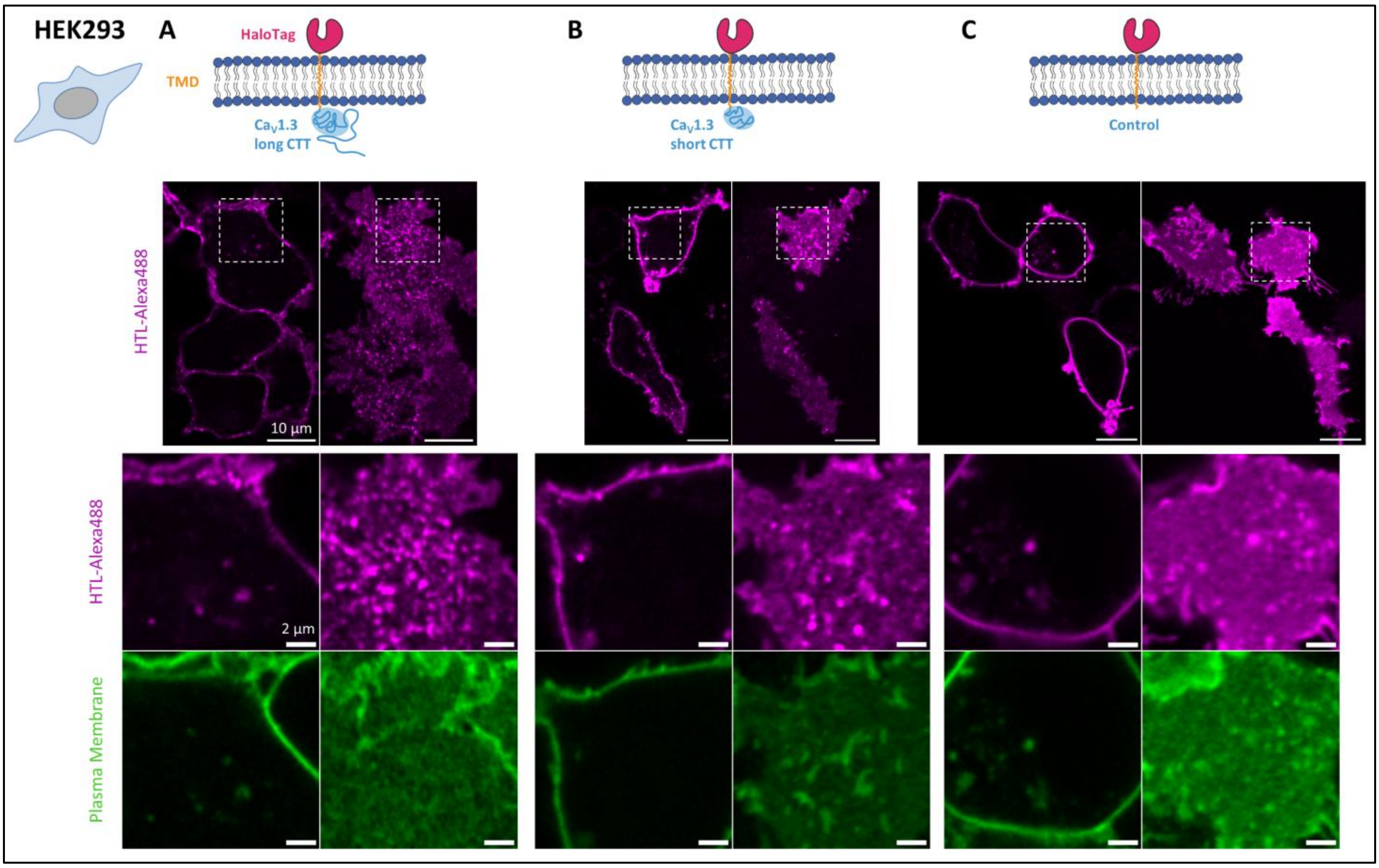

A) $Ca_V1.3$ C-terminal cytosolic tail (CTT, long isoform) fused to cell-surface HaloTag was expressed in HEK293 CT6232 cells and labeled with cell-impermeable HTL-Alexa488. The cells were co-stained with the plasma membrane marker Cholesterol-PEG-KK114 and imaged by live-cell confocal microscopy.

B) Expression of the equivalent fusion protein containing the short C-terminal tail splice variant.

C) Expression of a control construct containing only cell-surface HaloTag without CTT sequence, serving as a negative control.

Figure S8: Illustration of the custom-built optical setup (described in the Methods section)

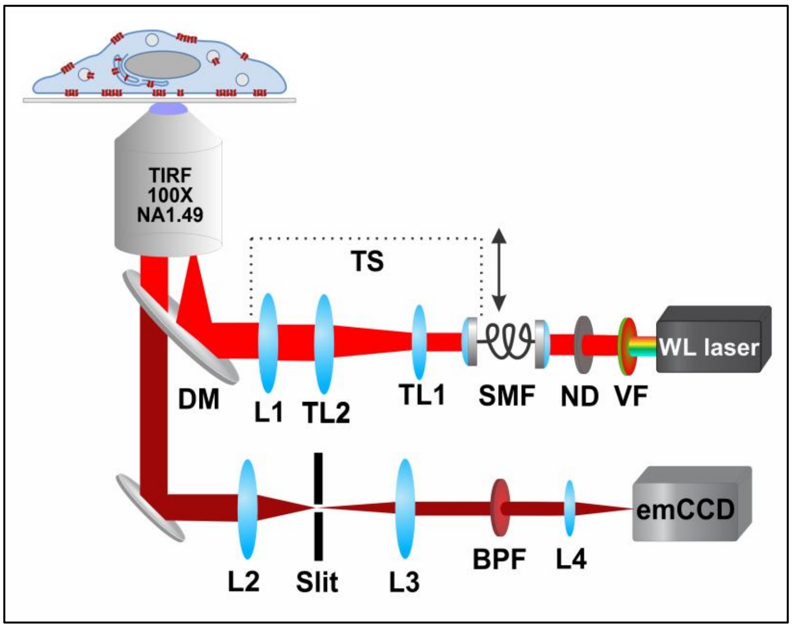

Supplement: Supplementary file 1 — Supplementary material 1 [file mmc1.pdf]
